# Book Notices

**Published:** 1879-06-20

**Authors:** 


### *BOOK NOTICES.*

*LECTURES ON ELECTRICITY IN ITS RELATIONS TO MEDICINE AND SURGERY.* By A. D. ROCKWELL, M.D., Electro-Therapeutist to the New York State Woman's Hospital, Member of the American Neurological Association, etc., etc.—New York: Wm. Wood, 27 Great Jones street.

The increasing interest in Electro-Physics of late will doubtless cause this and other works on the subject to be sought for. Electricity as a remedial agent has been so little understood and practiced by the profession, that the great body of them are wholly uninformed on the subject. This work being plain and practical, if read by the practitioner, would be likely to awaken an interest in this important and much neglected field of study.

*RHYMES OF SCIENCE, WISE AND OTHERWISE, WITH ILLUSTRATIONS.*—New York Industrial Publication Company.

This is a collection of humorous articles in rhyme with comic illustrations, calculated to amuse and interest the reader, especially one of scientific proclivities.

*ATLAS OF SKIN DISEASES.* By LOUIS A. DUHRING, M.D., Prof. of Skin Diseases in the Hospital of the University of Pennsylvania; Physician to the Dispensary for Skin Diseases, Philadelphia; Dermatologist to the Philadelphia Hospital, etc. Part v.: Lippincott & Co., Philadelphia—illustrating *Scabies, Herpes Zoster, Tinea, Sycosis and Eczema.*

We feel that we cannot too highly commend the above work to the profession—to the practitioner not less than to the student of medicine.

No department of medical science is so much neglected and so little understood as that of dermatology. The illustrations in the above work are so life-like, so admirably executed and so beautiful that the reader cannot fail to be charmed, interested and instructed. Such a work must tend greatly to relieve dermatology of the dryness and intricacy usually attached to it by the student, and impart to it an impulse of interest and attraction hitherto unknown.

*THE NATIONAL DISPENSATORY:* Containing the Natural history, pharmacy, actions and uses of medicines, including those recognised in the pharmacopeas of the U. S. and Great Britain. By ALFRED STILLE, M.D., L.L.D., Prof. of the Theory and Practice of Medicine and of Clinical Medicine in the University of Pennsylvania, and John M. Maish, Ph. D., Prof. of Materia Medica and Botany in the Philadelphia College of Pharmacy. H. C. Lea, Philadelphia.

This is a royal octavo of over sixteen hundred pages, illustrated by numerous wood-cuts. The book is admirably gotten up, the type is distinct, and the drugs being alphabetically arranged makes it a most acceptable and convenient book for reference. The whole design, plan and details of the work is useful, practical and comprehensive, presenting a vast array of facts and information, including late and important additions to the Materia Medica, and furnishing the student and the practitioner not only a clear description of remedial agents, both mineral and vegetable, but discussing also their medicinal properties with that force, clearness and precision which the author's great ability and experience so well fits him to perform.

It has been said that the discoveries and advances in therapeutics in the U. S. has surpassed in the last decade those of any other nation, and so rapid and vast has been the accumulation of new agents in this department, and so varied and wonderful the properties ascribed to the new remedies, that the practitioner is troubled to keep up with these discoveries, and is bewildered with the multiplicity and the properties ascribed to them. To have this vast field of matter reviewed by such a man as Prof. Stille, the chaff sifted out and the pure grain presented to the profession, is certainly a matter for gratulation, and one which the profession will doubtless appreciate. The work contains a very full general index, and, also an index of therapeutics, with tables of weights and measures, table of maximum and minimum doses, and other valuable information.

*A CHEMICAL TREATISE ON DISEASES OF THE LIVER.* By Dr. FRIED. THEOD. FRERICHS, Prof. of Chemical Medicine in the University of Berlin, etc.; Medical Privy-Counsellor and Medical Adviser to the Ministry of Public Instruction and Medicine at Berlin. In three volumes. Translated by Chas. Murcheson, M.D., F. R. C. P., Physician to the London Fever Hospital; Lecturer on Pathological Anatomy, etc.—New York: Wm. Wood & Co.

The above is the last of a work of three volumes first published on *Diseases of the Liver* by the author, each containing about 240 octavo pages, neatly gotten up and beautifully illustrated, representing many phases of morbid anatomy with remarks of chemical and practical nature highly useful and interesting. Every practitioner would do well to secure the three volumes of this valuable work, now complete. With all that has been said and written of the liver, few physicians know as much about it as they ought to know, and many who suppose themselves well posted in this class of affections will be surprised at the many useful facts and practical items found in this admirable work.

*TRANSACTIONS OF THE AMERICAN GYNECOLOGICAL SOCIETY.* Vol. 3 for the year 1878. Boston: Houghton, Osgood & Co.

"This volume contains the index to the gynecological and obstetric literature of all countries for the year 1877, prepared with the co-operation of Dr. J. S. Billings, U. S. A., in charge of the National Medical Library in Washington."

It is a volume of over 400 pages, illustrated and containing able and interesting papers by the following writers: Drs. Wm. Goodell, J. C. Reeve, Marion Sims, J. P. White, J. T. Johnson, T. A. Emmet, H. P. C. Wilson, R. A. F. Penrose, W. H. Byford, W. L. Richardson, S. C. Busey, H. F. Garrigues, A. H. Smith, H. F. Campbell, T. Parvin, J. E. Taylor, E. Van de Warker, A. R. Jackson, Fordyce Barker, T. M. Drysdale, Nathan Bozeman.

*POTT'S DISEASE—ITS PATHOLOGY AND MECHANICAL TREATMENT, WITH REMARKS ON ROTARY LATERAL CURVATURE.* By Newton M. Shaffer, M.D., Surgeon in charge of the New York Orthopædic Dispensary, Orthopædic Surgeon to St. Luke's Hospital, N. Y.

This is an interesting monograph of 82 pages, illustrated, on the subject of Pott's disease, in which the writer takes issue with Dr Sayre on some points. It will repay perusal.

*POSOLOGICAL TABLE INCLUDING ALL THE OFFICINAL AND THE MOST FREQUENTLY EMPLOYED UNOFFICINAL PREPARATIONS.* By CHAS. RICE, Chemist, Department of Public Charities and Correction, New York, etc. William Wood & Co., 27 Great Jones street, New York.

This is a work of great utility to the practitioner, as it gives the correct name and average doses of medicines. It gives also the equivalents of weights and measures, common and decimal, which is specially important at this time by reason of the transition now going on from the apothecaries to the French decimal or metric system.

